# A Simple Yet Reliable 12S rRNA-Based Molecular Approach for Identifying Bat Species

**DOI:** 10.3390/ani15243643

**Published:** 2025-12-18

**Authors:** Subarna Barua, Asfiha Tarannum, Charles E. Rupprecht, Molly C. Simonis, Daniel Felipe Barrantes Murillo, Janna R. Willoughby, Chengming Wang

**Affiliations:** 1 Department of Pathobiology, College of Veterinary Medicine, Auburn University, Auburn, AL 36849, USA; szb0116@auburn.edu (S.B.); azt0147@auburn.edu (A.T.); mcs0252@auburn.edu (M.C.S.); 2 College of Forestry, Wildlife & Environment, Auburn University, Auburn, AL 36849, USA; 3 Department of Veterinary Pathobiology, College of Veterinary Medicine, Oklahoma State University, Stillwater, OK 74078, USA

**Keywords:** bat species identification, 12S rRNA, cytochrome c oxidase subunit I (COI), morphological identification, phylogeny

## Abstract

Bats (*Chiroptera*) represent nearly one-fifth of all mammalian species and play vital ecological roles as pollinators, pest controllers, and reservoirs of zoonotic pathogens. Accurate identification of bat species is essential for biodiversity monitoring, conservation, and disease surveillance. Traditional methods based on morphology or acoustic calls are often limited by overlapping features, while DNA barcoding using the cytochrome oxidase I (*COI*) gene can be hindered by sequence variability. In this study, we developed a simple, single-step PCR assay targeting a short, variable region of the mitochondrial 12S rRNA gene. Alignment of sequences from 232 bat species allowed the design of a single primer pair producing a 203–224 bp amplicon that successfully distinguished all species analyzed. The assay achieved 100% amplification success across 241 bat samples, with 97.2% concordance between molecular and morphological identification. Two samples showed sequence divergence suggestive of an undescribed species. Overall, ten bat species from six genera were identified, with *Eptesicus fuscus* being the most frequent. This assay offers a practical and robust approach for bat identification, supporting biodiversity assessment and pathogen surveillance in ecological and public health research.

## Simple Summary

Bats are fascinating animals that help nature by eating insects, spreading seeds, and pollinating plants. They also sometimes carry pathogens that can affect humans and other animals, so it is important to know exactly which bat species are present in an area. However, many bats look very similar, making it difficult to tell them apart just by appearance. In this study, we developed an easy DNA test that can accurately identify bat species from very small or even slightly damaged samples. The test looks at a short piece of the bat’s genetic material and works well for different species of bats. When we tested bats from several species, the method correctly identified every sample and even suggested that two bats might belong to a new, undescribed species. This test is simple and relies on standard laboratory tools and can help scientists and conservation workers identify bats more easily and better understand their role in ecosystems and disease ecology.

## 1. Introduction

Bats (order *Chiroptera*) comprise approximately 20% of all mammalian species, with over 1500 recognized species globally [1,2,3]. They exhibit remarkable behavioral and morphological diversity and are increasingly recognized as bioindicators of ecosystem health [4,5], providers of critical ecosystem services such as pollination and pest control [6], and markers of conservation-priority areas [7]. However, at least 16% of bat species face extinction due to human-driven threats, including urbanization, habitat loss, invasive species, and roost disturbance [8,9]. Many species also remain poorly studied, with limited data on their populations and biology [10,11,12].

Bats are increasingly recognized as key reservoir hosts for numerous viruses capable of causing severe zoonotic diseases in humans and animals [13,14,15]. Approximately 70% of emerging infectious diseases are zoonotic in origin [16], and bats have been implicated in several major outbreaks, including rabies [17,18] and coronaviruses with confirmed spillover potential, such as severe acute respiratory syndrome coronavirus (SARS-CoV, 2003) [19] and Middle East respiratory syndrome coronavirus (MERS-CoV, 2012) [20,21,22]. These dual ecological and epidemiological roles underscore the need for accurate and efficient species-level identification to support ecological research, conservation planning, and zoonotic disease surveillance.

Bats also pose unique challenges to researchers due to their nocturnal behavior, flight capability, migratory habits, small body size, and cryptic morphology [23,24]. Accurate taxonomic resolution is essential for distinguishing morphologically similar species, identifying individuals at roost, and mapping species distributions [25,26]. Traditional identification methods, including morphological analysis and acoustic monitoring each have notable limitations. Morphological assessments rely on diagnostic features such as cranial structure, dentition, or baculum morphology [27] but are generally restricted to deceased or well-preserved specimens and are unreliable for juveniles or damaged individuals. In live specimens, overlapping or variable morphological traits can lead to misidentification [28]. Acoustic monitoring offers a non-invasive alternative but often fails to detect low-intensity callers, cannot easily distinguish species with overlapping call signatures, and depends on comprehensive regional call libraries [29,30].

Molecular methods have become the cornerstone of bat species identification, typically targeting mitochondrial loci such as cytochrome b (*cyt b*) [31], cytochrome c oxidase subunit 1 (*cox1*) [7,32,33,34,35], or segments of the hypervariable domain II within the mitochondrial control region (D-loop) that capture intraspecific diversity [36,37]. Among these, the cytochrome c oxidase subunit I gene (*COI*) has become the universal barcode for animals [38,39] and performs well in bats due to its low intraspecific and high interspecific variability [40], making it frequently applied in taxonomic and biodiversity studies [35,41,42,43].

Nevertheless, COI-based barcoding in bats faces challenges. The bat mitochondrial genome exhibits accelerated evolution and deep intraspecific divergences [40], which can lead to paraphyly and inconsistent species boundaries. Designing universal COI primers across *Chiroptera* is difficult due to high sequence diversity, often requiring taxon-specific primers and limiting large-scale or field-based applications.

Here, we present a simple and reliable single-reaction PCR assay targeting the mitochondrial 12S rRNA gene for bat species identification. The 12S rRNA region combines high interspecific variability with conserved primer-binding sites, enabling amplification across diverse bat lineages. This assay provides broad taxonomic coverage and robust amplification from a single primer set, making it suitable for laboratory and field applications, analyses of degraded samples, and high-throughput biodiversity assessments. The proposed 12S rRNA-based approach complements existing *COI*-based methods by offering a practical, broadly accessible alternative for accurate bat species identification.

## 2. Materials and Methods

### 2.1. Bats

A total of 265 bat carcasses, obtained from the Alabama Department of Public Health between 2023 and 2025, were included in this study. The brain and brainstem of each bat were tested for rabies prior to collection, and only rabies-negative individuals were included in the analyses. Twenty-four bats were excluded due to carcass autolysis.

### 2.2. DNA Extraction of Muscle Tissue from Bats

Approximately 40 mg of muscle tissue was collected from each bat and placed in a 2 mL microcentrifuge tube containing 300 µL of 1× PBS. Samples were homogenized with three zirconia beads using a Precellys 24 homogenizer (Bertin Instruments, Montigny-le-Bretonneux, France) for two cycles of 30 s at 5000 rpm, with a 120 s interval between cycles. Following homogenization, 200 µL of each homogenate was processed for nucleic acid extraction using the IndiMag 2 automated magnetic-bead system (INDICAL Inc., Orlando, FL, USA) with prefilled reagent cartridges, according to the manufacturer’s instructions. Genomic DNA was eluted in 100 µL of elution buffer and stored at −20 °C until further analysis.

### 2.3. Design of Primers to Amplify Bat Species

All available mitochondrial sequences for bat species, with emphasis on those present in North America, were retrieved from GenBank and aligned using Vector NTI v11.5 (Invitrogen, Carlsbad, CA, USA) and MEGA 12.1 (ClustalW, Paris, France). The sequences used for primer design represented 232 bat species across 20 families and 179 genera (Table 1; Supplementary File S1). Primers (sense: 5′-GGTAAATYTCGTGCCAGCCACC; antisense: 5′-AAGCATAGTGGGGTATCTAATCCCAGTTT-3′) were designed to target conserved regions across all bat species, while the resulting amplicon regions are highly polymorphic.

PCR assays were conducted using a LightCycler^®^ 96 real-time PCR system (Roche Diagnostics, Indianapolis, IN, USA) following previously described protocols [44]. In brief, PCR was performed in a 20 µL reaction containing 10 µL of extracted DNA, 5× PCR FRET buffer, 400 µM dNTPs, 0.34 U Platinum Taq polymerase, and 1 µM of each primer. Thermal cycling consisted of 18 high-stringency step-down cycles (6 × 95 °C for 15 s/75 °C for 60 s; 9 × 95 °C for 15 s/73 °C for 60 s; 3 × 95 °C for 15 s/71 °C for 30 s/72 °C for 30 s), followed by 25 relaxed-stringency fluorescence-acquisition cycles (95 °C for 15 s; 58 °C for 8 s; 65 °C for 30 s; 72 °C for 30 s).

The sensitivity of the assay was verified using one gBlock gene fragment containing the PCR amplicon of *Eptesicus fuscus* (Integrated DNA Technologies, Coralville, IA, USA). Based on the molecular weight of the gBlock fragment, 10-fold serial dilutions ranging from 10^4^ to 10^0^ copies per 10 µL reaction were prepared in triplicate to determine the detection limit. Quantitative standards consisted of plasmid-cloned target DNA at five concentrations (10,000, 1000, 100, 10, and 1 copies). These standards and sterile water negative control were included in every PCR assay.

**Figure 1:**
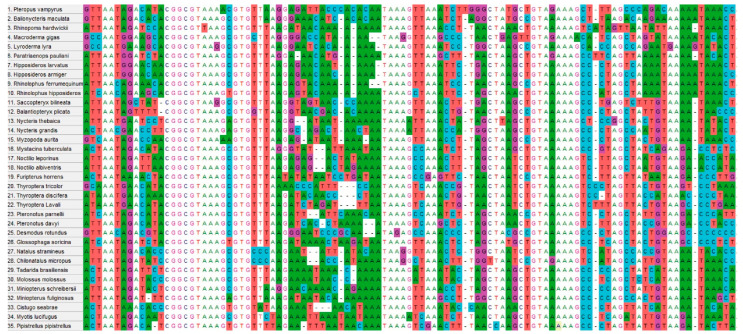
Nucleotide sequences representing 35 bat species for 20 families were amplified by the established PCR in this study. The amplified regions exhibit high polymorphism, with no identical sequences observed among the bat species analyzed. The bat species shown in this image correspond to those listed in Table 2 and illustrated in Figure 2.

**Figure 2:**
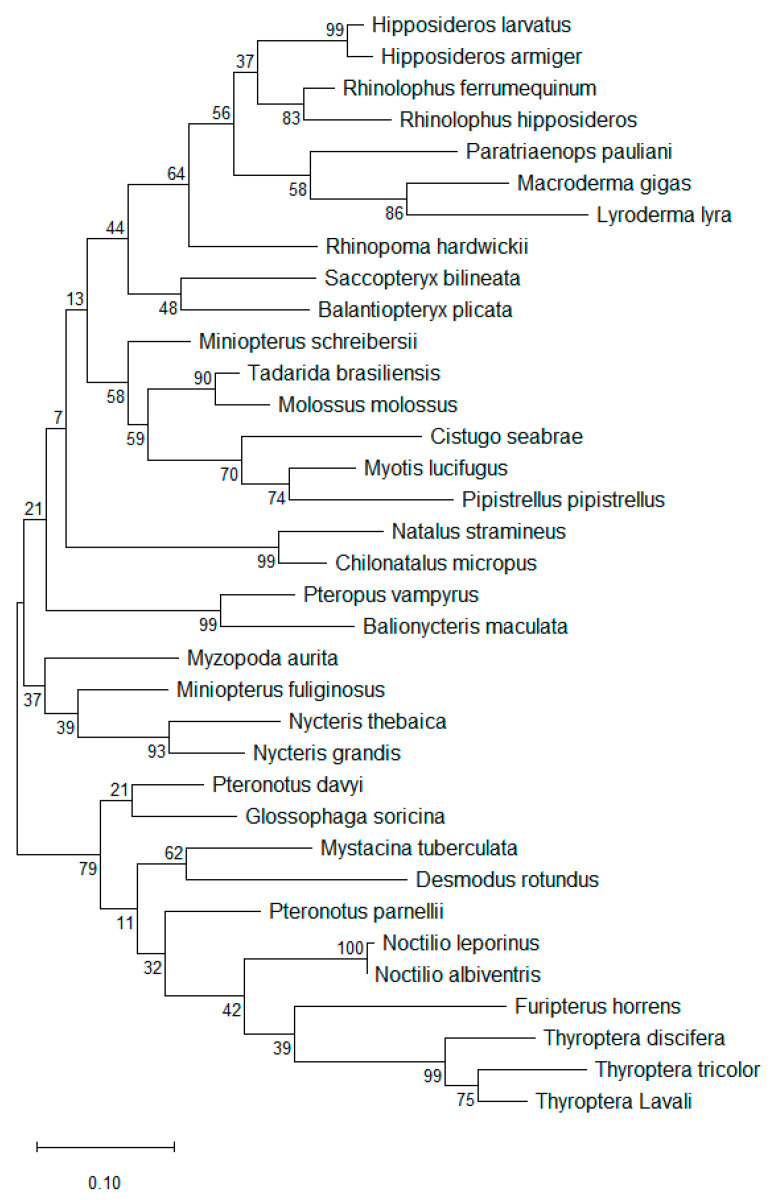
**Phylogenetic tree for 35 representative bat species in 20 families.** Evolutionary relationships were inferred using the Maximum Likelihood method based on nucleotide substitutions, and the tree with the highest log likelihood is shown [45]. The bat species depicted correspond to those listed in Table 2 and illustrated in Figure 1. The phylogenetic analysis reveals substantial genetic diversity among all bat species, with *Noctilio leporinus* and *Noctilio albiventris* differing by a single nucleotide and exhibiting the highest sequence similarity (100%) in this dataset.

PCR products were submitted to ELIM Biopharmaceuticals (Hayward, CA, USA) for bidirectional Sanger sequencing. Obtained nucleotide sequences were compared to existing genomes using BLASTn (https://blast.ncbi.nlm.nih.gov/Blast.cgi?PROGRAM=blastn&BLAST_SPEC=GeoBlast&PAGE_TYPE=BlastSearch, accessed on 9 November 2025). Phylogenetic analysis of all 232 bat species was performed, including calculation of pairwise p-distances (Supplementary File S2). In addition, a simplified phylogenetic tree and the corresponding amplicon regions were generated for 35 representative sequences, comprising one to two species per family across the 20 families (Figure 1 and Figure 2). The p-distance was calculated as the number of differing positions between two sequences divided by their sequence length, for all 232 bat species (Supplementary File S3) and for the 35 representative species (Table 2).

### 2.4. Morphological Identification of Bats

To compare molecular and morphological identification, 71 bat carcasses were identified to species using published identification keys [28]. For specimens that were challenging to identify, particularly dehydrated carcasses or skulls dissected for rabies testing, key morphological traits and comparative methods were applied. Briefly, *Myotis* spp. were distinguished based on the presence or absence of a keeled calcar, plagiopatagia connections to the hindlimb, relative length of hindlimb toe hairs, and tragus characteristics. *Lasiurus seminolus* and *Lasiurus borealis* were differentiated by direct comparison of fur coloration. For species with overlapping morphometric measurements, such as *Nycticeius humeralis* and *Eptesicus fuscus*, identification was based on the presence or absence of a keeled calcar (*Eptesicus fuscus*, keeled; *Nycticeius humeralis*, unkeeled).

## 3. Results

The sensitivity of this established PCR in this study was found to be 10 copies of the target gene per reaction. The alignment of nucleotide sequences and testing of different regions and primers led to the design of a single primer set that is highly conserved across all 232 bat species included in the design of the primers. The short amplicon region between these primers (203–224 nucleotides) is highly polymorphic among all bat species, as demonstrated by phylogenetic trees (Figure 2, Supplementary File S2), nucleotide alignments (Figure 1), and the P distance calculations (Table 2, Supplementary File S3). Within this short amplicon region, no two of the 232 bat species showed identical nucleotide sequences.

**Figure 3:**
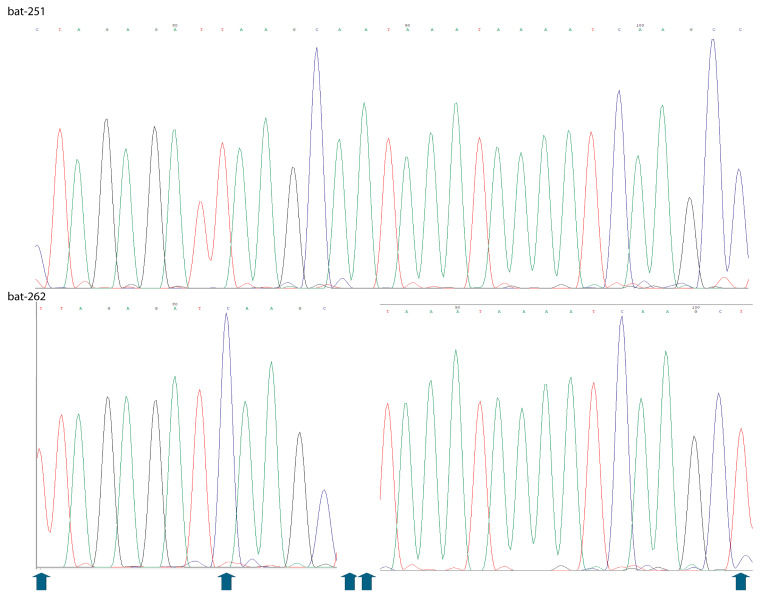
**Nucleotide sequence divergence between two *Myotis grisescens* identified by morphological characteristics.** Two bats (bat-262 and bat-251) were morphologically identified as *Myotis grisescens*. Comparative analysis of their chromatogram sequences revealed a 4.5% nucleotide divergence (10 of 221 nucleotides), consisting of 8 single-nucleotide mismatches and two deletions. Molecular identification determined these two bats are *Myotis austroriparius* and *Myotis dominicensis*, respectively. Arrows indicate the nucleotide mismatches between the two sequences. The sequence shown for bat-251 is continuous and contains two mismatches and two deletions relative to that of bat-262.

Among the 241 bats tested in this study, the molecular identification method successfully identified 100% of individuals (241/241), yielding high-quality Sanger sequencing results as shown in Figure 3.

When the molecular approach was applied for bat species identification, 99.6% of the bats (240/241) exhibited at least 97.1% nucleotide similarity to identified reference species (Table 3), whereas most other identified species demonstrated approximately 99% nucleotide similarity. One bat, identified as *Lasiurus ega*, showed the lowest similarity at 90.7% (Table 3).

Of the 71 bats identified using both molecular and morphological methods, 97.2% (69/71) showed consistent results between the two approaches. For the two bats with differing identifications (Table 3), molecular analysis indicated they were *Myotis austroriparius* and *Myotis dominicensis*, each showing only two nucleotide differences compared to their respective reference sequences. Morphological identification classified both bats as *Myotis grisescens*, while the 12S rRNA sequence for *Myotis grisescens* was unavailable. While morphological identification determined two bats as *Myotis grisescens* with confidence, they exhibited 4.5% nucleotide divergence in the 12S rRNA gene (Figure 3). Future work will involve collaborating with reference laboratories and wildlife agencies to generate a verified 12S rRNA and COI reference sequences for *M. grisescens*, which will allow us to determine whether the observed divergence reflects natural intraspecific variation or potential misidentification.

Overall, ten bat species across six genera were identified in this study, with *Eptesicus fuscus* being the dominant species (113/241, 46.9%) (Table 3). Nine of these ten species, except *Lasiurus ega*, have been previously reported in Alabama, USA.

## 4. Discussions

This study establishes a simple, accurate, and reliable molecular method for identifying bat species using the mitochondrial 12S rRNA gene. The assay was designed to be broadly applicable across *Chiroptera*, and our results demonstrate consistent performance across 20 of the 21 recognized bat families. The 12S rRNA fragment provided clear sequence differentiation among all analyzed species, with no instances of identical sequences between species from different families, highlighting its strong resolving power for species-level identification.

The robustness of this method derives from the genetic properties of the 12S rRNA region, which combines conserved primer-binding sites, allowing efficient amplification across diverse taxa, with variable internal regions that capture interspecific divergence. The single-reaction format further enhances reproducibility and accessibility, making this method practical for laboratories with varying technical capacities, including those in field or resource-limited settings.

Compared to the commonly used *COI* gene [41,42,46], the 12S rRNA assay offers several practical advantages. The COI amplicons are usually longer (~650 bp), which can be problematic when DNA is degraded, as in field-collected tissues, guano, or museum specimens. Universal COI primers often fail to amplify efficiently across all bat taxa, requiring multiple taxon-specific primer sets [47,48], limiting their routine use in large-scale surveys. In contrast, the 12S assay’s shorter amplicon and universal primer design in this study and COI mini-barcode [35] enable high amplification success and sequencing accuracy, even from low-quality or mixed DNA templates [43]. Interestingly, the BLASTn results and sequence alignments showed that the amplicon region of the established 12S rRNA system contains six nucleotide differences between *Myotis thysanodes* (GenBank accession MN299336) and *M. evotis* (KC747659), both of which share the same COI mini-barcode. This makes the method complementary to both morphological identification and COI-based barcoding: morphology remains essential for field identification and voucher confirmation, while COI continues to provide phylogenetic and evolutionary insights. In this study, molecular and morphological identifications were concordant in 97.2% of cases. Interestingly, two bats identified morphologically as *Myotis grisescens* exhibited a 4.5% nucleotide divergence in 12S rRNA, unusually high for mammals, where ribosomal genes typically show >99% sequence identity between individuals of the same species [49,50].

The southern yellow bat (*Lasiurus ega*) is widely distributed from southern Texas through eastern Mexico and Central America, extending across much of tropical South America to northern Argentina [51,52]. In this study, BLASTn analysis of one sample revealed only 90.7% similarity to the closest known *Lasiurus ega* sequence, which has not been reported in Alabama, suggesting these individuals may represent an undescribed species.

The availability of 12S rRNA reference sequences is critical for this approach. Many bat species also remain poorly studied, with limited data on their populations and biology [10,11]. Expanding the database to include broader geographic and taxonomic coverage will improve accuracy and utility for global biodiversity assessments. Integration with COI and nuclear markers will further enhance species resolution and elucidate cryptic lineages. As anticipated from the conserved nature of rRNA genes, the established platform is not exclusive to bats and can amplify DNA from other vertebrate species, but not from arthropods.

## 5. Conclusions

The 12S rRNA-based assay provides a practical, accurate, and universally applicable tool for bat species identification. It complements morphological and COI-based methods, enabling reliable species confirmation, biodiversity surveys, and pathogen surveillance. Its simplicity, robustness, and broad applicability make it especially valuable for ecological, conservation, and public health studies, even in laboratories with standard PCR and sequencing capabilities. Importantly, this method is straightforward to implement, requiring only standard PCR and sequencing equipment, making it accessible to virtually any laboratory and a powerful tool for routine species identification and biodiversity monitoring.

## Figures and Tables

**Table 1:** Bat species (*n* = 232) from 20 families and 179 genera included to design primers in this study.

Family	Genus and Species	Family	Genus and Species
Cistugidae	*Cistugo seabrae*	Pteropodidae	*Desmalopex leucopterus*
Emballonuridae	*Balantiopteryx plicata*		*Dyacopterus spadiceus*
	*Cormura brevirostris*		*Desmalopex leucopterus*
	*Diclidurus scutatus*		*Dyacopterus spadiceus*
	*Paremballonura atrata*		*Eidolon helvum*
	*Peropteryx kappleri*		*Eonycteris spelaea*
	*Rhynchonycteris naso*		*Epomophorus gambianus*
	*Saccopteryx bilineata*, *S. leptura*		*Epomops franqueti*
	*Taphozous nudiventris*		*Haplonycteris fischeri*
Furipteridae	*Furipterus horrens*		*Harpyionycteris celebensis*
Hipposideridae	*Aselliscus stoliczkanus*		*Hypsignathus monstrosus*
	*Doryrhina cyclops*		*Latidens salimalii*
	*Hipposideros abae*, *H. armige*, *H. larvatus*		*Macroglossus sobrinus*
	*Macronycteris commersonii*		*Megaerops niphanae*
Megadermatidae	*Lyroderma lyra*		*Megaloglossus woermanni*
	*Macroderma gigas*		*Melonycteris melanops*
Miniopteridae	*Miniopterus fuliginosus*, *M. inflatus*, *M. pusillus*, *M. schreibersii*		*Mirimiri acrodonta*
Molossidae	*Cynomops mexicanus*		*Myonycteris angolensis*
	*Eumops auripendulus*, *E. perotis*		*Nanonycteris veldkampii*
	*Molossus molossus*		*Neopteryx frosti*
	*Mops condylurus*		*Nesonycteris fardoulisi*
	*Nyctinomops femorosaccus*, *N. macrotis*		*Notopteris macdonaldii*
	*Otomops martiensseni*		*Nyctimene albiventer*, *N. cephalotes*
	*Ozimops planiceps*		*Paranyctimene raptor*
	*Promops centralis*		*Penthetor lucasii*
	*Sauromys petrophilus*		*Plerotes anchietae*
	*Tadarida brasiliensis*, *T. teniotis*		*Ptenochirus wetmorei*
Mormoopidae	*Mormoops blainvillii*, *M. megalophylla*		*Pteralopex taki*
	*Pteronotus davyi*, *P. gymnonotus*, *P. parnellii*, *P. personatus*		*Pteropus medius*, *P. rayneri*, *P. vampyrus*
Mystacinidae	*Mystacina tuberculata*		*Rousettus amplexicaudatus*
Myzopodidae	*Myzopoda aurita*		*Sphaerias blanfordi*
Natalidae	*Chilonatalus micropus*		*Stenonycteris lanosus*
	*Natalus macrourus*, *N. stramineus*, *N. tumidirostris*		*Styloctenium wallacei*
Noctilionidae	*Noctilio albiventris. N. leporinus*		*Syconycteris australis*
Nycteridae	*Nycteris grandis*, *N. thebaica*		*Thoopterus nigrescens*
Phyllostomidae	*Ametrida centurio*	Rhinolophidae	*Rhinolophus ferrumequinum*, *R. hipposideros*, *R. luctus*, *R. pusillus*, *R. sinicus*
	*Anoura caudifer*	Rhinonycteridae	*Paratriaenops pauliani*
	*Ardops nichollsi*	Rhinopomatidae	*Rhinopoma hardwickii*
	*Ariteus flavescens*	Thyropteridae	*Thyroptera discifera*, *T. lavali*, *T. tricolor*
	*Artibeus jamaicensis*, *A. lituratus*	Vespertilionidae	*Aeorestes cinereus*
	*Brachyphylla cavernarum*		*Alionoctula coromandra*
	*Carollia brevicauda*		*Antrozous pallidus*
	*Centurio senex*		*Arielulus circumdatus*
Phyllostomidae	*Chiroderma trinitatum*		*Barbastella barbastellus*
	*Choeroniscus minor*		*Bauerus dubiaquercus*
	*Chrotopterus auritus*	Vespertilionidae	*Cassistrellus dimissus*
	*Desmodus rotundus*		*Chalinolobus tuberculatus*
	*Diaemus youngi*		*Cnephaeus hottentotus*
	*Diphylla ecaudata*		*Corynorhinus mexicanus*, *C. rafinesquii*, *C. townsendii*
	*Ectophylla alba*		*Dasypterus ega*, *D. intermedius*, *D. Xanthinus*
	*Enchisthenes hartii*		*Eptesicus fuscus*, *E. nilssonii*
	*Erophylla sezekorni*		*Euderma maculatum*
	*Gardnerycteris crenulatum*		*Eudiscopus denticulus*
	*Glossophaga morenoi*, *G. soricina*		*Glauconycteris beatrix*
	*Glyphonycteris daviesi*		*Glischropus bucephalus*
	*Hsunycteris thomasi*		*Hesperoptenus blanfordi*
	*Hylonycteris underwoodi*		*Histiotus macrotus*
	*Lampronycteris brachyotis*		*Hypsugo alaschanicus*
	*Leptonycteris curasoae*, *L. nivalis*, *L. yerbabuenae*		*Ia io*
	*Lichonycteris obscura*		*Idionycteris phyllotis*
	*Lionycteris spurrelli*		*Kerivoula minuta*
	*Lonchorhina aurita*		*Laephotis namibensis*
	*Lophostoma brasiliense*		*Lasionycteris noctivagans*
	*Macrophyllum macrophyllum*		*Lasiurus blossevillii*, *L. borealis*, *L. seminolus*
	*Macrotus californicus*, *M. waterhousii*		*Mimetillus moloneyi*
	*Mesophylla macconnelli*		*Murina hilgendorfi*
	*Mimon bennettii*		*Murina huttoni rubella*
	*Monophyllus redmani*		*Myotis austroriparius*, *M. californicus*, *M. ciliolabrum*, *M. evotis*, *M. keenii*, *M. leibii*, *M. lucifugus*, *M. septentrionalis*, *M. thysanodes*, *M. velifer*, *M. volans*
	*Musonycteris harrisoni*		*Neoromicia somalica*
	*Phylloderma stenops*		*Nyctalus aviator*
	*Phyllostomus hastatus*		*Nycticeinops eisentrauti*
	*Platyrrhinus brachycephalus*		*Nycticeius humeralis*
	*Pygoderma bilabiatum*		*Nyctophilus geoffroyi*
	*Rhinophylla pumilio*		*Otonycteris hemprichi*
	*Sphaeronycteris toxophyllum*		*Parastrellus hesperus*
	*Stenoderma rufum*		*Perimyotis subflavus*
	*Sturnira magna*		*Philetor brachypterus*
	*Tonatia saurophila*		*Pipistrellus pipistrellus*
	*Trachops cirrhosus*		*Plecotus auritus*
	*Trinycteris nicefori*		*Pseudoromicia brunnea*, *P. rendalli*
	*Uroderma bilobatum*		*Rhogeessa aenea*, *R. alleni*
	*Vampyriscus brocki*		*Scotoecus hirundo*
	*Vampyrodes caraccioli*		*Scotomanes ornatus*
	*Vampyrum spectrum*		*Scotonycteris bergmansi*
Pteropodidae	*Acerodon celebensis*, *A. jubatus*		*Scotophilus viridus*
	*Aethalops alecto*		*Submyotodon latirostris*
	*Alionycteris paucidentata*		*Thainycteris aureocollaris*
	*Aproteles bulmerae*		*Tylonycteris fulvida*
	*Balionycteris maculata*		*Vansonia rueppellii*
	*Boneia bidens*		*Vespadelus darlingtoni*
	*Casinycteris campomaanensis*		
	*Chironax melanocephalus*		
	*Cynopterus brachyotis*		

**Table 2:** The p-distance between the 35 representative bat species in 20 families.

Pteropus_vampyrus																																		
Balionycteris_maculata	0.13																																	
Rhinopoma_hardwickii	0.25	0.21																																
Macroderma_gigas	0.27	0.26	0.23																															
Lyroderma_lyra	0.26	0.21	0.23	0.18																														
Paratriaenops_pauliani	0.25	0.24	0.18	0.19	0.23																													
Hipposideros_lanvatus	0.22	0.21	0.15	0.22	0.19	0.17																												
Hipposideros_armiger	0.20	0.20	0.17	0.22	0.19	0.19	0.03																											
Rhinolophus_ferumequinum	0.22	0.18	0.15	0.20	0.19	0.20	0.11	0.12																										
Rhinolophus_hipposideros	0.23	0.23	0.16	0.20	0.21	0.21	0.13	0.13	0.08																									
Saccopteryx_bilineata	0.24	0.23	0.18	0.26	0.23	0.19	0.19	0.20	0.22	0.24																								
Balantiopteryx_plicata	0.25	0.24	0.19	0.24	0.23	0.19	0.19	0.18	0.17	0.20	0.16																							
Nycteris_thebaica	0.28	0.27	0.21	0.24	0.22	0.23	0.19	0.20	0.22	0.22	0.21	0.21																						
Nycteris_grandis	0.24	0.24	0.22	0.26	0.21	0.26	0.18	0.20	0.20	0.22	0.23	0.22	0.12																					
Myzopoda_aurita	0.19	0.22	0.21	0.25	0.23	0.21	0.18	0.18	0.17	0.18	0.20	0.18	0.17	0.19																				
Mystacina_tuberculata	0.24	0.26	0.23	0.27	0.25	0.25	0.23	0.23	0.23	0.22	0.25	0.23	0.26	0.24	0.23																			
Noctilio_leoprinus	0.26	0.26	0.18	0.27	0.27	0.22	0.20	0.19	0.20	0.18	0.21	0.19	0.23	0.23	0.20	0.19																		
Noctilio_albiventris	0.26	0.26	0.18	0.27	0.27	0.22	0.20	0.19	0.20	0.18	0.21	0.19	0.23	0.30	0.19	0.19	0.01																	
Furipterus_horrens	0.35	0.33	0.27	0.34	0.30	0.25	0.24	0.25	0.27	0.28	0.29	0.28	0.34	0.32	0.30	0.22	0.21	0.21																
Thyroptera_tricolor	0.33	0.34	0.30	0.33	0.28	0.30	0.28	0.29	0.30	0.32	0.30	0.31	0.27	0.24	0.25	0.26	0.24	0.24	0.26															
Thyroptera_discifera	0.29	0.30	0.24	0.30	0.30	0.26	0.21	0.22	0.25	0.26	0.26	0.26	0.26	0.25	0.25	0.24	0.21	0.21	0.23	0.14														
Thyroptera_Lavali	0.31	0.33	0.27	0.35	0.30	0.27	0.24	0.26	0.27	0.28	0.25	0.27	0.25	0.25	0.25	0.22	0.20	0.20	0.22	0.10	0.11													
Pteronotus_parnellii	0.25	0.26	0.21	0.29	0.25	0.25	0.23	0.24	0.21	0.21	0.24	0.25	0.25	0.24	0.17	0.14	0.16	0.16	0.19	0.20	0.24	0.19												
Pteronotus_davyi	0.24	0.26	0.19	0.27	0.25	0.21	0.20	0.20	0.20	0.18	0.23	0.21	0.22	0.21	0.18	0.16	0.17	0.17	0.22	0.20	0.20	0.17	0.12											
Desmodus_rotundus	0.30	0.29	0.31	0.31	0.29	0.31	0.30	0.27	0.26	0.26	0.27	0.24	0.28	0.27	0.23	0.21	0.23	0.23	0.30	0.30	0.30	0.22	0.22	0.21										
Glossophaga_sonicina	0.25	0.27	0.20	0.25	0.25	0.20	0.20	0.21	0.22	0.19	0.22	0.21	0.20	0.21	0.18	0.20	0.19	0.18	0.24	0.25	0.19	0.20	0.17	0.12	0.21									
Natalus_stramineus	0.26	0.24	0.24	0.27	0.27	0.25	0.21	0.20	0.23	0.22	0.25	0.25	0.25	0.25	0.23	0.26	0.23	0.24	0.32	0.30	0.25	0.28	0.24	0.20	0.31	0.25								
Chilonatalus_micropus	0.25	0.26	0.24	0.30	0.29	0.26	0.25	0.22	0.27	0.25	0.22	0.22	0.28	0.27	0.27	0.27	0.21	0.21	0.30	0.34	0.29	0.31	0.24	0.24	0.29	0.27	0.10							
Tadanda_brasiliensis	0.22	0.23	0.17	0.22	0.19	0.19	0.16	0.16	0.15	0.14	0.18	0.18	0.18	0.22	0.17	0.18	0.20	0.20	0.27	0.29	0.25	0.27	0.21	0.18	0.25	0.18	0.22	0.22						
Molossus_molossus	0.22	0.21	0.18	0.24	0.21	0.19	0.18	0.18	0.16	0.16	0.16	0.20	0.21	0.25	0.20	0.18	0.18	0.18	0.26	0.31	0.25	0.28	0.23	0.19	0.26	0.19	0.21	0.21	0.05					
Miniopterus_schreibersii	0.20	0.19	0.16	0.23	0.22	0.19	0.18	0.18	0.15	0.17	0.18	0.18	0.16	0.19	0.16	0.21	0.18	0.18	0.29	0.26	0.24	0.24	0.21	0.18	0.26	0.17	0.21	0.20	0.11	0.13				
Miniopterus_fuliginosus	0.20	0.20	0.16	0.24	0.21	0.20	0.18	0.19	0.17	0.20	0.17	0.18	0.17	0.15	0.16	0.25	0.21	0.21	0.31	0.30	0.27	0.28	0.23	0.19	0.25	0.17	0.23	0.21	0.15	0.18	0.10			
Cistugo_seabrae	0.28	0.27	0.20	0.28	0.25	0.27	0.21	0.22	0.19	0.20	0.26	0.26	0.25	0.23	0.26	0.24	0.25	0.25	0.26	0.25	0.21	0.23	0.24	0.23	0.31	0.25	0.25	0.28	0.15	0.17	0.20	0.23		
Myotis_lucifugus	0.25	0.25	0.21	0.27	0.27	0.25	0.23	0.24	0.21	0.23	0.22	0.25	0.23	0.22	0.21	0.21	0.21	0.21	0.29	0.26	0.27	0.22	0.22	0.23	0.31	0.25	0.28	0.27	0.16	0.18	0.16	0.19	0.18	
Pipistrellus_pipistrellus	0.32	0.34	0.25	0.32	0.33	0.28	0.25	0.26	0.25	0.26	0.25	0.27	0.26	0.24	0.22	0.23	0.23	0.23	0.29	0.25	0.25	0.24	0.24	0.25	0.32	0.26	0.29	0.30	0.21	0.25	0.22	0.24	0.18	0.14

MEGA was performed to calculate the p-distance between 35 representative bat species. The p-distance is calculated as P (the number of positions at which two sequences differ)/L (the length of each of the two sequences).

**Table 3:** Ten bat species identified in this study.

Species Identified	Number of Bats (%)	Identification Method	Nucleotide Similarity (%)
*Eptesicus fuscus*	113 (46.9%)	Molecular and morphological	98.9–100.0%
*Lasiurus borealis*	31 (12.9%)	Molecular and morphological	98.1–100.0%
*Lasiurus ega* *	1 (0.4%)	Molecular only	90.7%
*Lasiurus seminolus*	9 (3.7%)	Molecular and morphological	99.1–100.0%
*Myotis austroriparius*	6 (2.5%)	Molecular and morphological	98.6–100.0%
*Myotis dominicensis*	6 (2.5%)	Molecular only	97.1–97.6%
*Myotis grisescens* **	2 (0.8%)	Morphological only	97.3–99.1%
*Nycticeius humeralis*	32 (13.3%)	Molecular and morphological	98.0–100.0%
*Pipistrellus subflavus*	3 (1.2%)	Molecular and morphological	99.0–100.0%
*Tadarida brasiliensis*	38 (15.8%)	Molecular and morphological	98.5–100.0%

* The BLASTn was performed using the regions between the upstream and downstream primers, which are shared by all bat species. ** The determination of the *Myotis grisescens* was based on morphological identification while the 12S rRNA gene for *Myotis grisescens* is not available.

## Data Availability

All data related to this work is included in this manuscript, including the Supplementary Files.

## Supplementary Materials

The following supporting information can be downloaded at: https://www.mdpi.com/article/10.3390/ani15243643/s1, File S1: List of 232 bat species across 20 families and 179 genera with GenBank accession numbers used for primer design in this study; File S2: Phylogenetic relationships among 232 bat species from 20 families. Evolutionary relationships were inferred using the Maximum Likelihood method based on nucleotide substitutions, and the tree with the highest log likelihood is presented. The bat species included correspond to those listed in Supplementary Files S1 and S3. The analysis reveals substantial phylogenetic diversity among all bat species, with no identical amplicon regions observed among them; File S3: Pairwise p-distance among 232 bat species across 20 families and 179 genera. Pairwise p-distances among 232 bat species were calculated using MEGA. The p-distance is defined as *P*/*L*, where Pis the number of nucleotide differences between two sequences and Lis the sequence length. The lowest observed p-distance was 0.006, and no identical amplicons were detected among the bat species. Reference [53] are cited in the Supplementary Materials.
