# Supplementary material for: A Simple Yet Reliable 12S rRNA-Based Molecular Approach for Identifying Bat Species
Source: Animals (Basel). 2025 Dec 18;15(24):3643. doi: 10.3390/ani15243643 (PMC12729680; doi:10.3390/ani15243643)
Supplement: Supplementary file 1 [file animals-15-03643-s001.zip › Legends for supplementary files.pdf]

Legend for three supplementary files

Supplementary File S1. List of 232 bat species across 20 families and 179 genera with GenBank accession numbers used for primer design in this study

Supplementary File S2. Phylogenetic relationships among 232 bat species from 20 families. Evolutionary relationships were inferred using the Maximum Likelihood method based on nucleotide substitutions, and the tree with the highest log likelihood is presented. The bat species included correspond to those listed in Supplementary Files S1 and S3. The analysis reveals substantial phylogenetic diversity among all bat species, with no identical amplicon regions observed among them.

Supplementary File S3. Pairwise p-distance among 232 bat species across 20 families and 179 genera. Pairwise p-distances among 232 bat species were calculated using MEGA. The p-distance is defined as P/L, where $P$is the number of nucleotide differences between two sequences and $L$is the sequence length. The lowest observed p-distance was 0.006, and no identical amplicons were detected among the bat species.
