# Supplementary figures and images for: A Simple Yet Reliable 12S rRNA-Based Molecular Approach for Identifying Bat Species
Source: Animals (Basel). 2025 Dec 18;15(24):3643. doi: 10.3390/ani15243643 (PMC12729680; doi:10.3390/ani15243643)

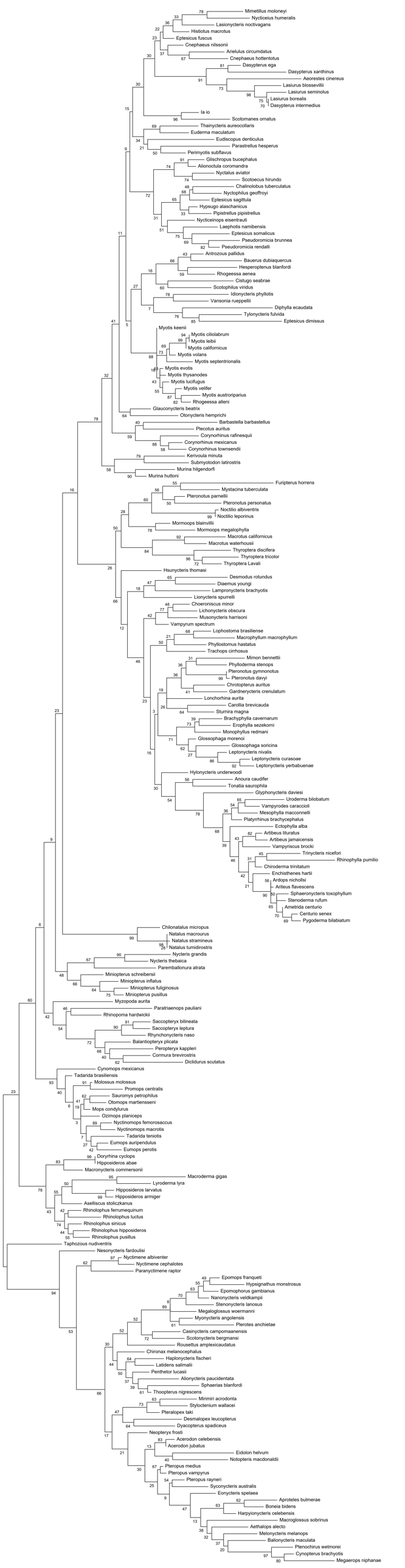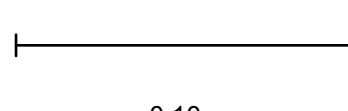

Supplement: Supplementary file 1 [file animals-15-03643-s001.zip › Supplementary File S2.pdf]
